# Differentiation of Induced Pluripotent Stem Cells to Lentoid Bodies Expressing a Lens Cell-Specific Fluorescent Reporter

**DOI:** 10.1371/journal.pone.0157570

**Published:** 2016-06-20

**Authors:** Taruna Anand, Thirumala R. Talluri, Dharmendra Kumar, Wiebke Garrels, Ayan Mukherjee, Katharina Debowski, Rüdiger Behr, Wilfried A. Kues

**Affiliations:** 1 Friedrich-Loeffler-Institut, Institut für Nutztiergenetik, Neustadt, Germany; 2 ICAR-National Research Center on Equines, Hisar, India; 3 ICAR-Central Institute for Research on Buffalos, Hisar, India; 4 Medical School Hannover, Hannover, Germany; 5 German Primate Center, Göttingen, Germany; University of Pécs Medical School, HUNGARY

## Abstract

Curative approaches for eye cataracts and other eye abnormalities, such as myopia and hyperopia currently suffer from a lack of appropriate models. Here, we present a new approach for *in vitro* growth of lentoid bodies from induced pluripotent stem (iPS) cells as a tool for ophthalmological research. We generated a transgenic mouse line with lens-specific expression of a fluorescent reporter driven by the *alphaA crystallin* promoter. Fetal fibroblasts were isolated from transgenic fetuses, reprogrammed to iPS cells, and differentiated to lentoid bodies exploiting the specific fluorescence of the lens cell-specific reporter. The employment of cell type-specific reporters for establishing and optimizing differentiation *in vitro* seems to be an efficient and generally applicable approach for developing differentiation protocols for desired cell populations.

## Introduction

Age-related cataracts are one of the most prevalent ocular conditions resulting from the failure of specific cell types and represent the major eye disease in humans [1]. But a systematic approach to study human cataracts is hampered by the lack of appropriate models [2]. Therefore, *in vitro* systems for studying lens formation and disease mechanisms represent an alternative for ophthalmological research.

The understanding of lens morphogenesis and the involved cellular and molecular events serves as key in defining the general mechanisms of cell specification and gaining a better understanding of lens function. The eye lens originates from a single progenitor lineage, which comprises both the posterior lens fiber cells and the anterior lens epithelial cells [2]. In mammals, the lens progenitor cells originate from a vesicle at the lens placode [3,4] and the lens fiber cells terminally differentiate to ultimately contributing to the three-dimensional structure of the lens. This includes a massive up-regulation of lens-specific genes, such as alpha- and beta-crystallins [5,6]. Expression of alphaA crystallin (*Cryaa*) is initiated in the cells of the inverting lens placode and later on is restricted to the lens [5,6]. The Cryaa represents 20–40% of the crystallin content in the lens [7–9], and a molecular understanding of its temporally and spatially regulated expression in the lens is an important issue of cellular differentiation in general. Knock-out or loss-of-function mutations of the *Cryaa* gene have been shown to result in the formation of cataracts [10,11] and in apoptosis of lens epithelial cells [12], clearly indicating its pivotal role for lens function. Genetic studies in humans suggested a causative correlation between *Cryaa* mutations and cataract formations [13–20]. Previously, embryonic stem (ES) cells have been used to differentiate into lentoid bodies [2,21] and retinal cells [21] *in vitro* by using co-culture techniques with stromal cells [21] and by sequential supplementation of the culture medium with Noggin, fibroblast growth factor 2 (FGF2) and Wnt-3a [2]. Induced pluripotent stem (iPS) cells were used to generate retinal pigmented epithelium [22–24] and recently, the generation of lens progenitor cells from iPS cells of cataract patients and healthy donors [25], and the derivation of corneal epithelial cells from human iPS cells was achieved [26, 27]. In addition, an iPS cell-based disease model for ectodermal dysplasia and impaired corneal differentiation has been described [28].

**Fig 1:**
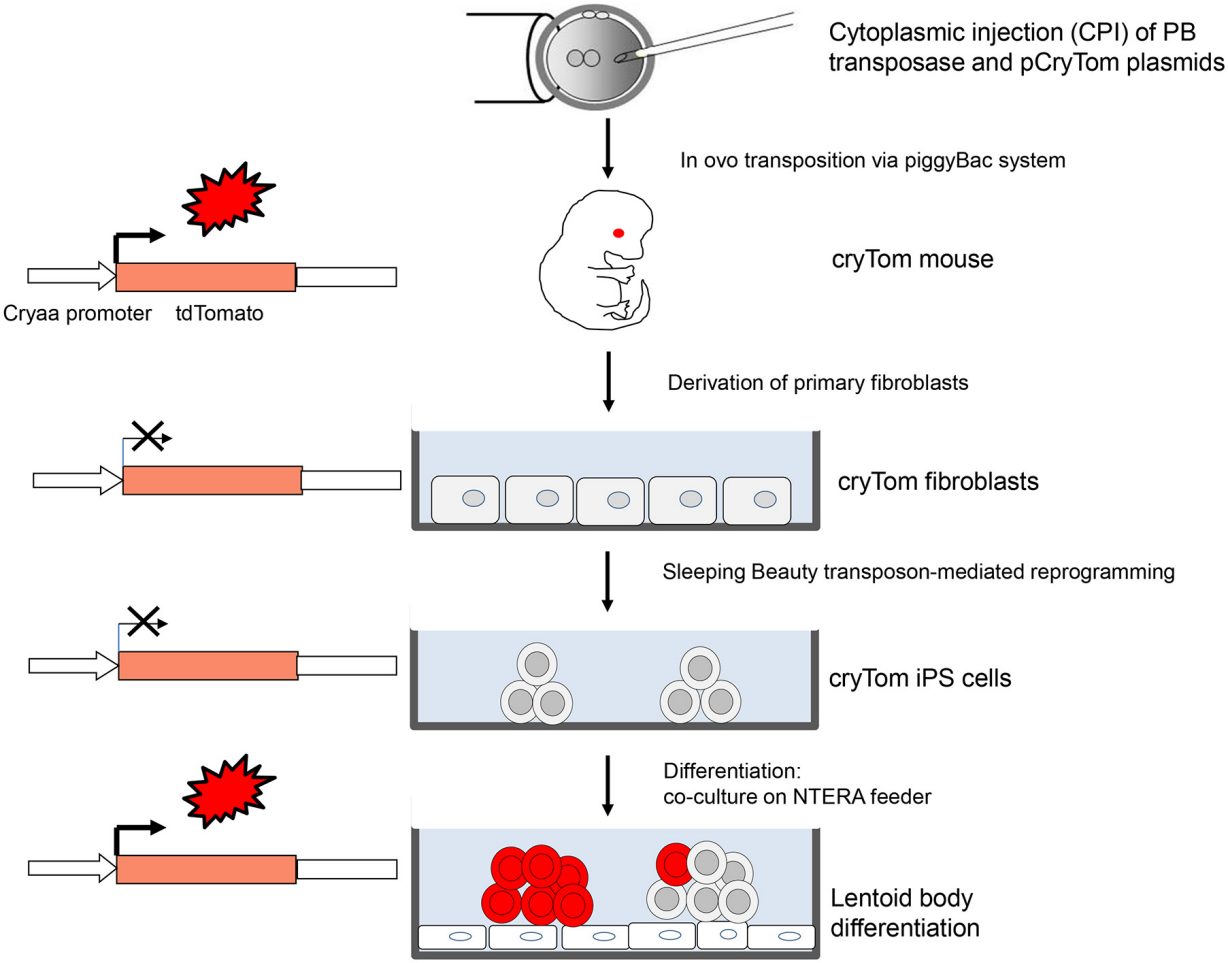
Schematic outline of reprogramming and programming to lens differentiation. The founder mouse was generated by *in ovo* transposition. Fibroblasts were isolated from a cryTom positive fetus at d11.5. At passage 2, the fibroblasts were reprogrammed to iPS cells by co-electroporation of a *Sleeping Beauty* reprogramming transposon and a SB expression plasmid as previously described [32, 49, 50]. The iPS cells were seeded on mitotically inactivated cell feeders (NTERA and P19), hypothesizing that the feeder will provide a niche for ectoderm and lens cell differentiation. Differentiation into lens cells should result in re-activation of the *Cryaa-tdTomato* reporter.

Here, we conducted a proof-of-principle study for the differentiation of murine iPS cells to lens cells. We exploited the cell-type specific expression of the *Cryaa* promoter for the generation of a transgenic mouse model with expression of a vital fluorophore reporter, tdTomato, in the eye lens. Fetal fibroblasts derived from these mice were reprogrammed to iPS cells, and the suitability of the reporter to follow differentiation into lens cells via lentoid body formation *in vitro* was assessed. We hypothesized that the derivation of iPS cells from a transgenic mouse line carrying the *Cryaa-tdTomato* construct can be used to follow differentiation into lens cells *in vitro* (Fig 1). This approach will facilitate the controlled development of more efficient protocols for lens cell-differentiation, and will aid to improve differentiation protocols with human cells.

## Results

### Generation and characterization of cryTom mouse line

**Fig 2:**
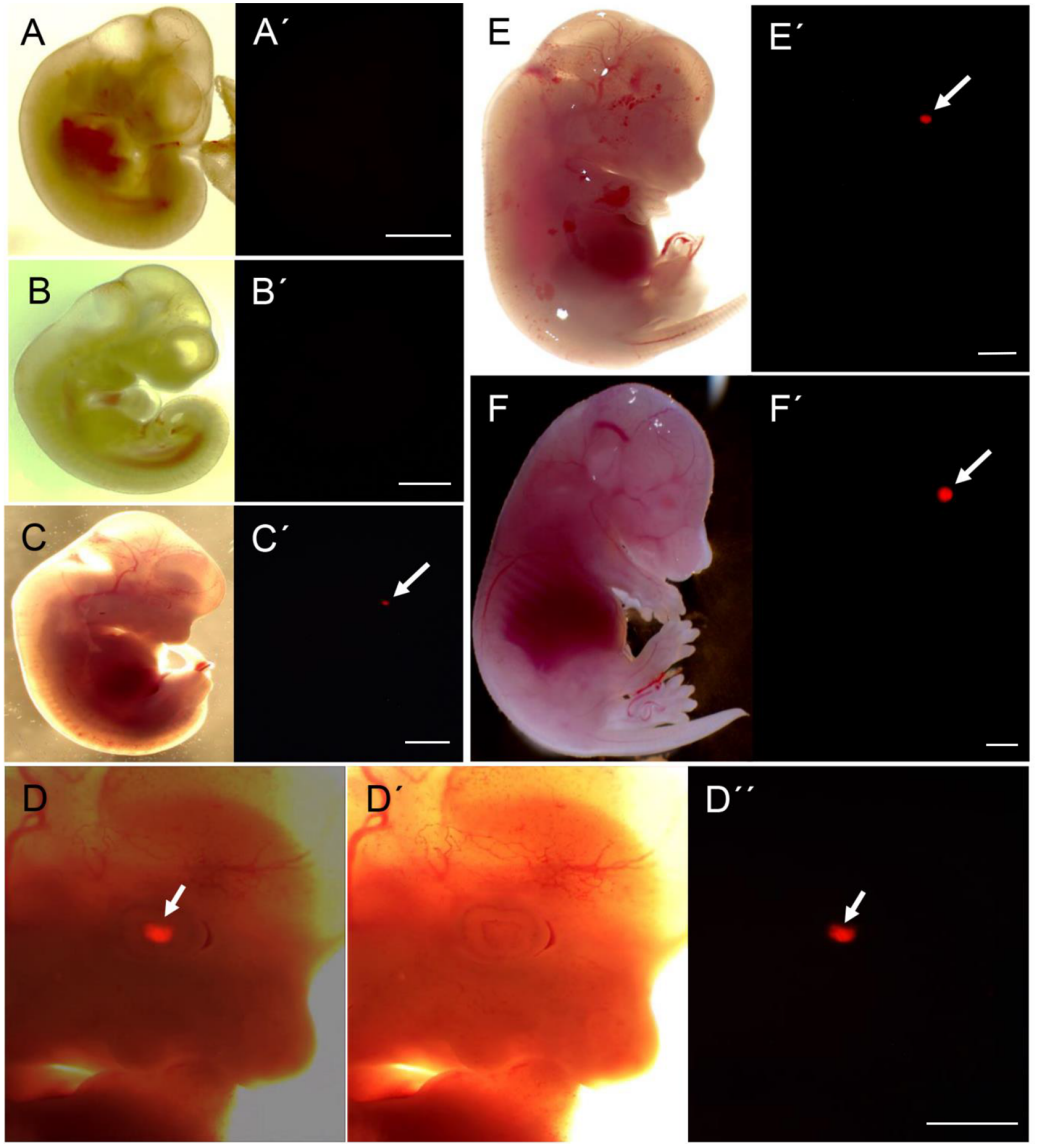
Exclusive expression in eye lens during fetal development. (A) Murine fetus at day 10.5 p.c., A´) corresponding fluorescence image. (B) Murine fetus at day 11.5 p.c., B´) corresponding fluorescence image. (C) Murine fetus at day 12.5 p.c., C´) corresponding fluorescence image, note the onset of tdTomato expression in the forming lens area (arrow). (D) Higher magnification of the d12.5 fetus, overlay, D´) brightfield and D”) fluorescence images. (E) Murine fetus at day 13.5 p.c., E´) corresponding fluorescence image. (F) Murine fetus at day 14.5 p.c., F´) corresponding fluorescence image. Size bars = 1 mm.

**Fig 3:**
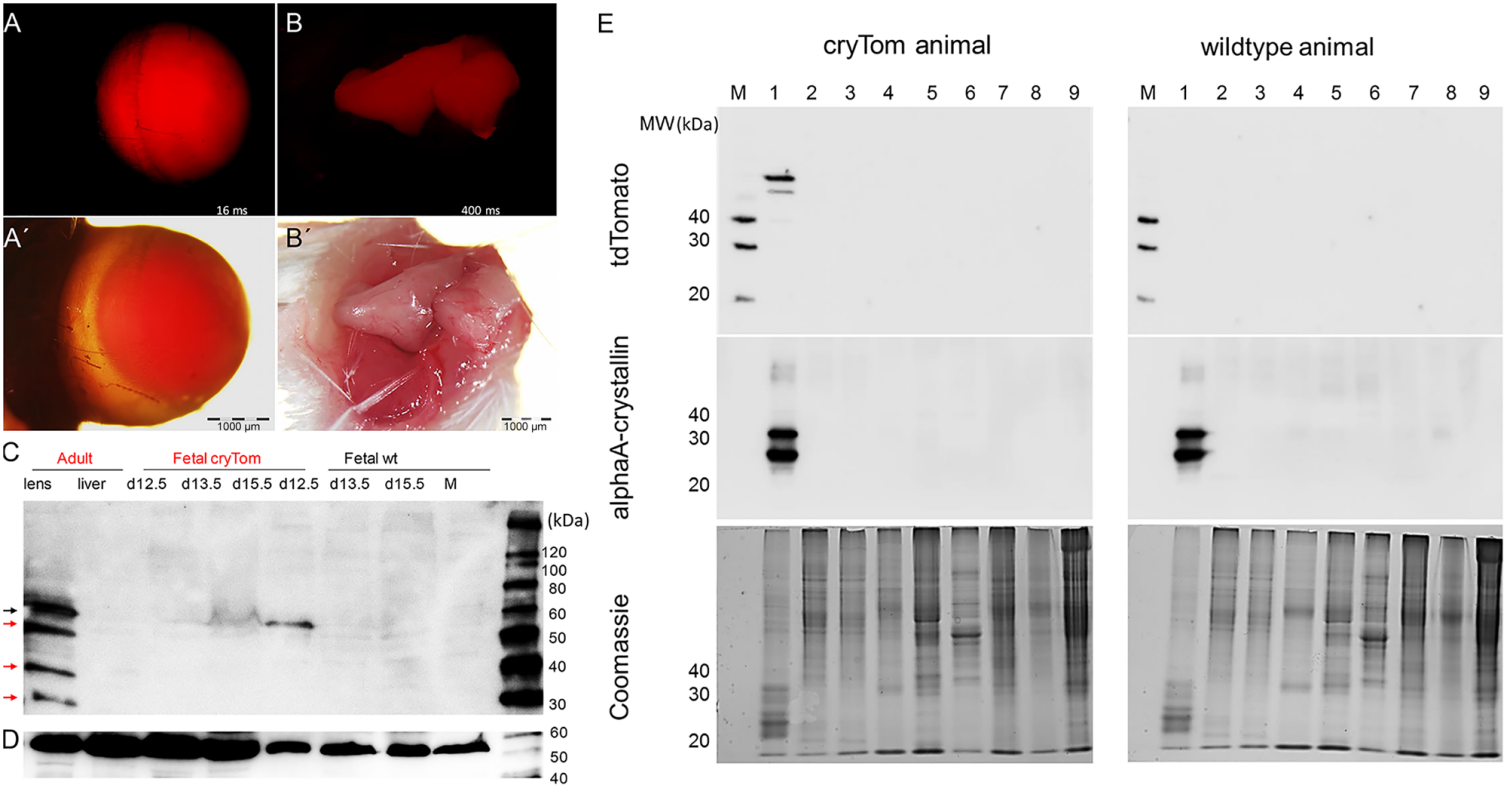
TdTomato expression in the adult eye. (A) TdTomato expression in the isolated mouse eye, A´) corresponding brightfield view, size bar = 1 mm. (B) TdTomato expression in ciliary muscle, B´) corresponding brightfield view, note the drastically increased exposure time relative to the lens to reveal expression in muscle, Size bar = 1 mm. (C) Immunoblot detection of tdTomato during prenatal stages, the full-sized tdTomato of about 54 kDa is detected (black arrow). In the adult lens several smaller degradation products are found (red arrows). M, molecular weight marker; cryTom, samples from transgenic animals and fetuses; wt, wildtype controls. (D) Western blotting of tubulin (loading control). (E) Expression of endogenous alphaA crystallin is similar in transgenic and wildtype animals. Top, Western blotting of tdTomato; bottom, Western blotting of endogenous alphaA crystallin with a polyclonal antibody; bottom, Coomassie stained gels as loading controls. M, molecular size marker; 1, eye lens; 2, ZNS; 3, cerebellum; 4, lung; 5, heart; 6, skel. muscle; 7, kidney; 8, skin; and 9, liver.

A *piggyBac* (PB) transposon (pCryTom) was designed, consisting of the *alphaA-crystallin* promoter, tdTomato cDNA and a SV40 poly adenylation sequence, flanked by PB inverted terminal repeats (ITR). Murine zygotes were treated by co-injection of pCryTom plasmid and pBP helper plasmid into the cytoplasm [29,30]. A total of 20 injected zygotes were transferred by surgical embryo transfer into the oviduct of one surrogate mother. One out of the delivered 8 pups was confirmed to carry the transposon construct by Southern blotting (S1 Fig). Importantly, the transgenic founder showed eye-specific expression of the tdTomato transposon (S1 Fig). The monomeric transposon was inherited in a Mendelian fashion and transgenic F1 and F2 offspring exhibited an identical phenotype (Figs 2 and 3).

During fetal development, the first expression of the reporter was found in the eye lens of day 12.5 fetuses (Fig 2), and lenses of older fetuses showed increasing fluorescence intensities. Likely, an increased cell number, increased expression level, and protein accumulation contributed to the increased reporter intensity. No ectopic expression of the reporter construct was found by whole mount imaging of fetuses, indicating that the reporter construct faithfully mirrors differentiation of the eye lens.

In accordance with the known expression pattern of the *Cryaa* gene ([5], www.genevestigator.com), the highest expression level of the cryTom was found in the adult eye lens, and reduced levels were detected in retina and ciliary muscle (Fig 3). In postnatal lens samples the full size tdTomato and three to four smaller products were consistently found in immunoblots using a polyclonal antibody (Fig 3). The smaller products most likely represent degradation products of tdTomato, which seemed to be removed at a slow rate in mature lenses. Western blotting of the endogenous alphaA crystallin showed that the expression of this lens protein was not affected by the transgenic status (Fig 3).

**Fig 4:**
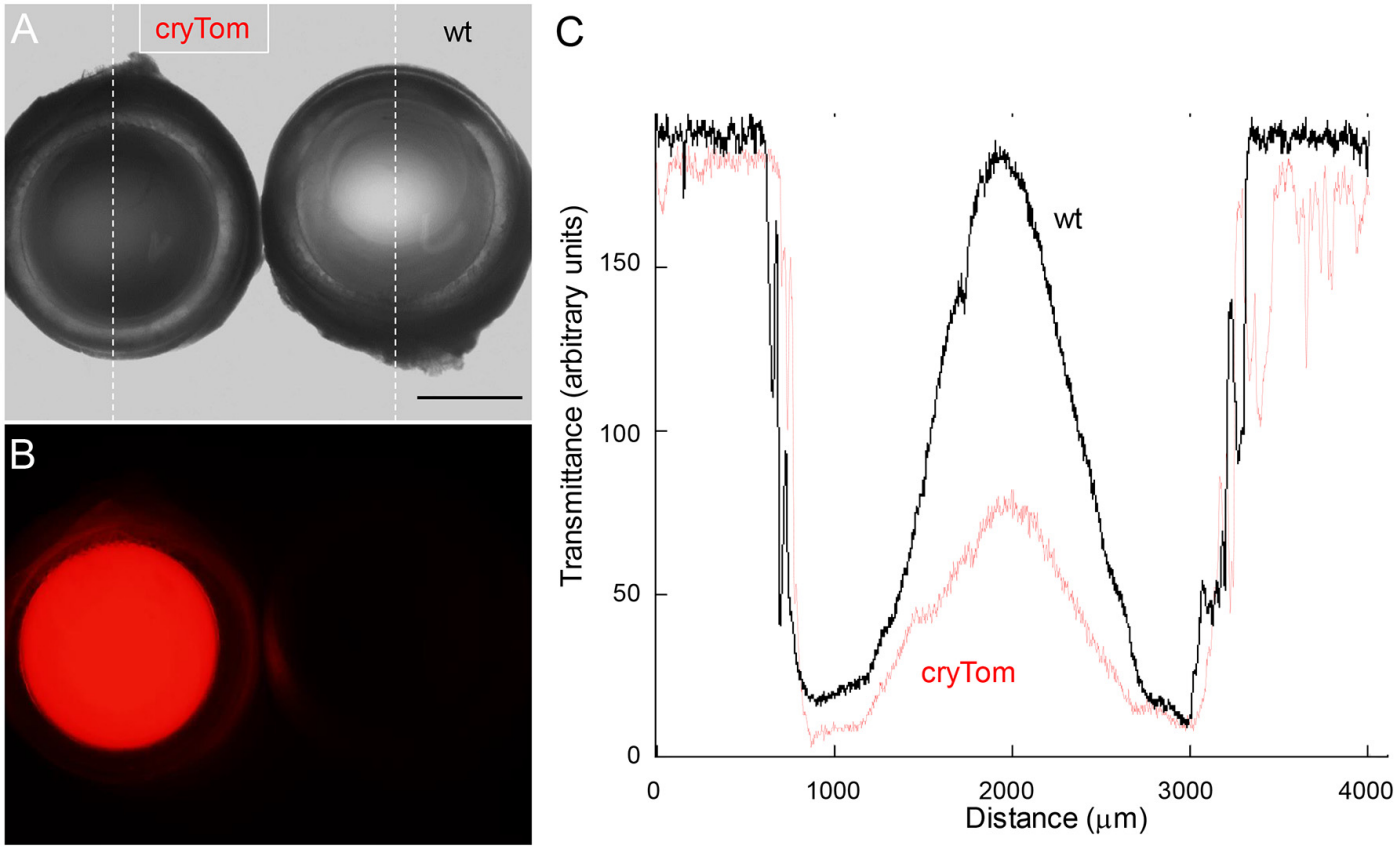
Reduced light transmission in tdTomato expressing lenses. (A) Isolated lenses from a cryTom and a wildtype animal are transmitted with white light from below, and grey scale images are recorded. The dotted lines indicate the measurement areas (see C), size bar = 1 mm. (B) Corresponding fluorescence image. (C) Quantification of light transmittance. Note that the wildtype lens (black line) shows almost complete light transmittance, whereas the cryTom lens (red line) shows a reduced transmittance. Measurement areas are the dotted lines indicated in A).

The apparent accumulation of smaller “degraded” products of tdTomato raised the question, whether the presence of these ectopic protein products may interfere with the highly ordered organisation of crystallin proteins in the lens. Therefore the light transmittance properties of wild type and cryTom lenses were assessed. Fig 4 shows a representative image of adult lenses recorded while illuminated from below in a stereomicroscope. A quantitative determination suggested that the transgenic lens indeed showed a reduced light transmittance in comparison to a non-transgenic lens.

### Reprogramming of cryTom fetal fibroblasts to induced pluripotent stem (iPS) cells

Heterozygous matings were initiated to isolate fetuses of day 11.5 of gestation, which were used to derive fetal fibroblasts. The fibroblasts were genotyped by PCR for the presence of the cryTom construct. As expected both cryTom-positive and cryTom-negative fibroblast populations did not express the reporter. Reprogramming of the fibroblasts to iPS was done by a non-viral approach employing a *Sleeping Beauty* (SB) transposon system as described previously [31,32]. Seven to nine days after co-electroporation of SB helper plasmid and multi-cistronic reprogramming transposon, encoding the murine cDNAs of *Oct4*, *Sox2*, *Klf4* and *c-Myc* separated by sequences coding for the self-cleaving 2A peptides, initial colonies appeared. Around day 18 post electroporation, individual colonies were picked and expanded. None of these iPS cultures did express the cryTom reporter, supporting the notion that lens-exclusive expression of cryTom is maintained under *in vitro* culture conditions.

**Fig 5:**
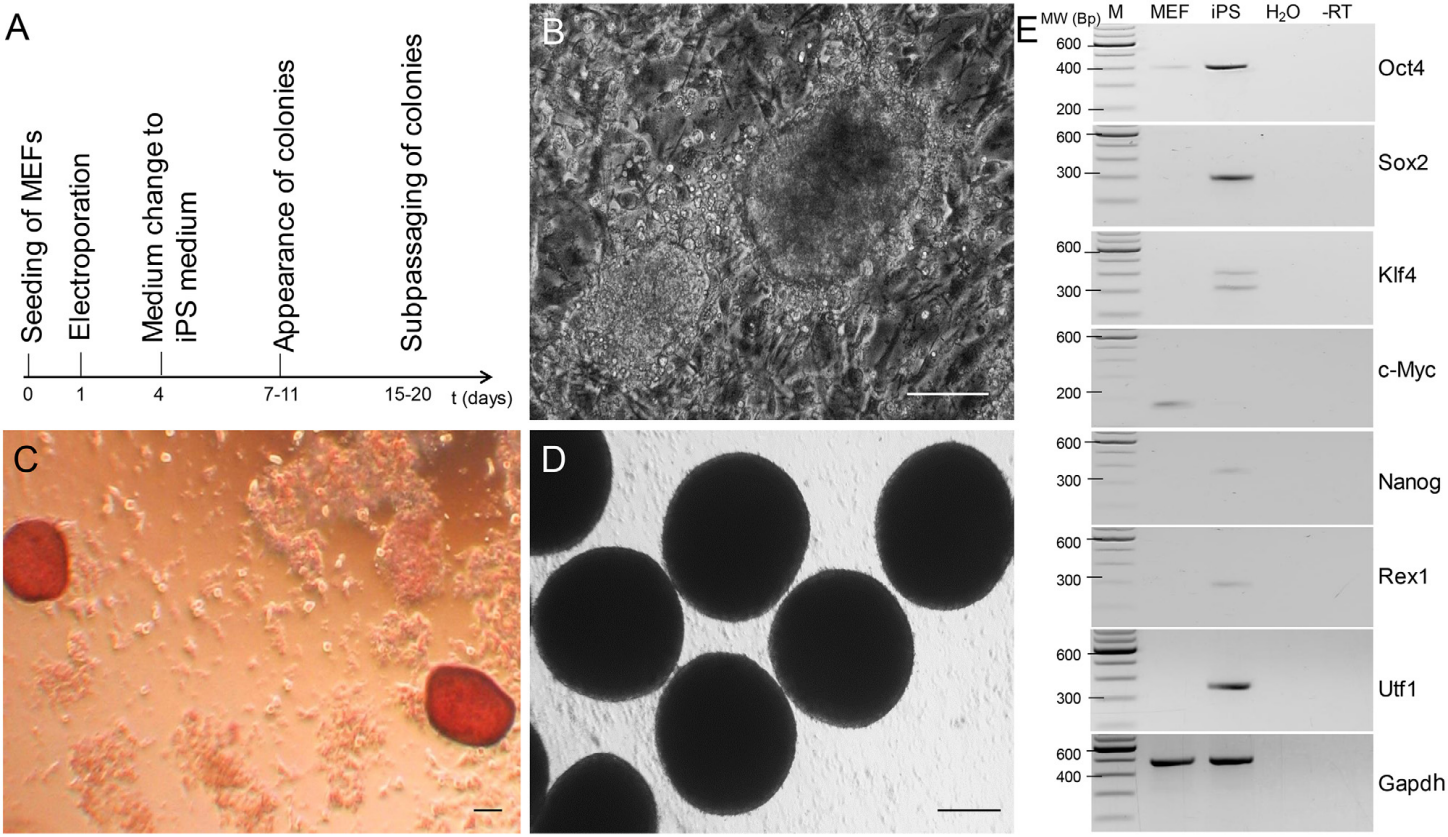
Characterization of murine iPS cells. (A) Schedule for non-viral iPS cell generation by SB transposon reprogramming [32, 49, 50]. (B) Initial colonies formed 9–15 days post electroporation. Bar = 20 micrometer. (C) AP stained culture 15 days post electroporation. Note, the intensively red stained colonies. Bar = 20 micrometer. (D) Upon culture in hanging drops, embryoid bodies formed readily. Bar = 50 micrometer. (E) Upregulation of stemness-related genes in the cryTom iPS cells.

The iPS cells expressed typical features of pluripotent cells (Fig 5). They were alkaline phosphatase positive and showed the typical colony growth of murine pluripotent cells. They showed an up-regulation of the stemness genes *Oct4*, *Sox2*, *Nanog*, *Utf2* and *Rex1*.

### Differentiation to lentoid bodies *in vitro*

Then we assessed, whether the cryTom construct can be utilized to establish and to follow differentiation into the lens cell lineage *in vitro*. Therefore mitotically-inactivated NTERA-2, P19 and STO cells were used as feeder cells, respectively. The NTERA-2 cells represent a committed human neuronal precursor line, and the P19 is a murine embryonic carcinoma cell line with differentiation potential into all three germ layers. We assumed that NTERA-2 and P19 may provide a suitable niche for differentiation of the iPSs towards the ectodermal direction, including lens cell differentiation. Critical factors for ectodermal differentiation may be surface epitopes and the secretion of paracrine factors, like BMP4. STO cells, a murine embryonic fibroblast line, served as control.

**Fig 6:**
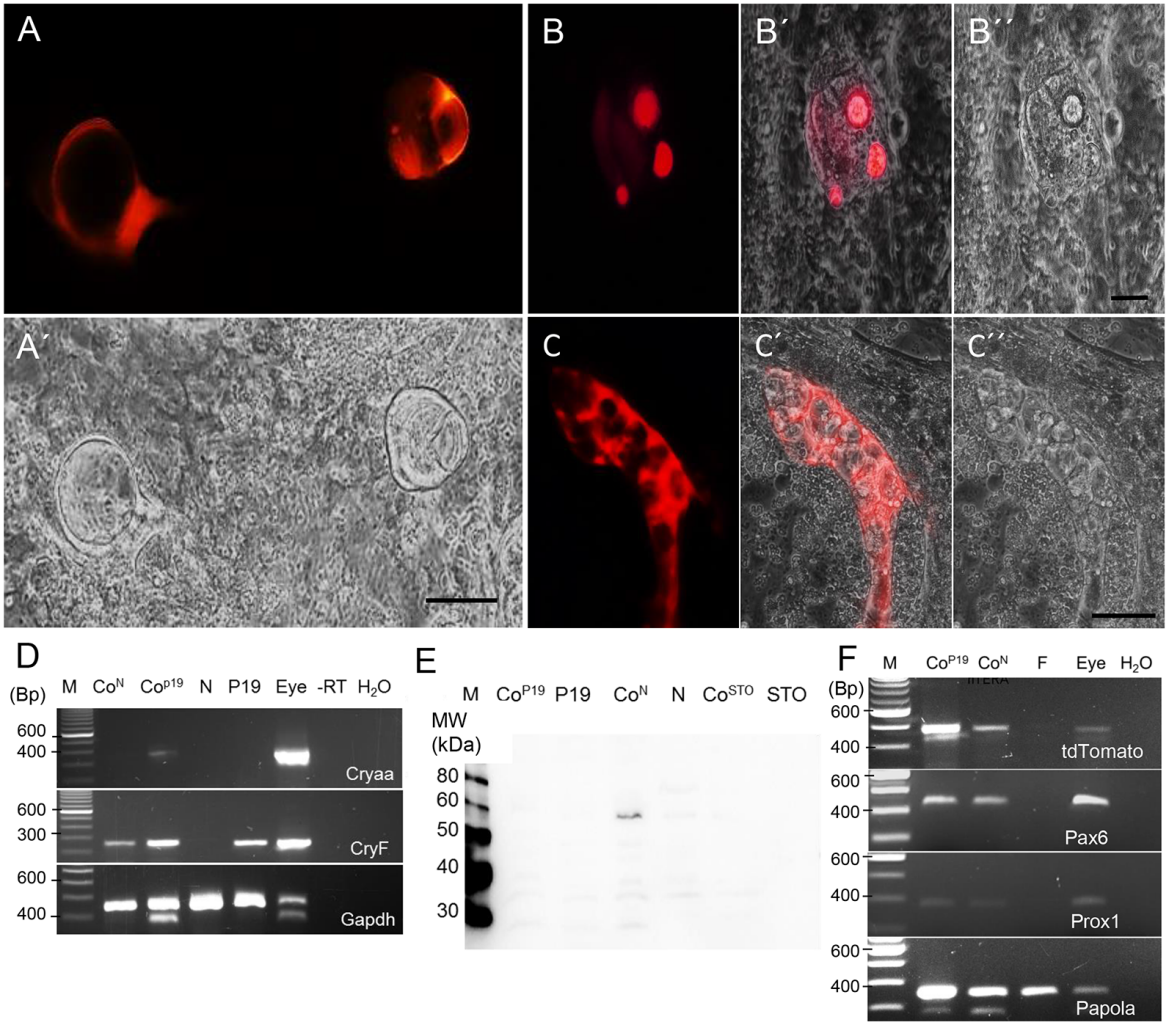
Characterization of *in vitro*-formed lentoid bodies. (A) Lentoid bodies with tdTomato expression derived from a co-culture of cryTom iPS on NTERA-2, A´) corresponding brightfield view. Bar = 50 micrometer. (B) Individual tdTomato-positive cells derived from a co-culture of cryTom iPS on P19, B´) overlay; B´´) corresponding brightfield view. Bar = 50 micrometer. (C) Lentoid body with tdTomato expression derived from a co-culture of cryTom iPS on P19, C´) overlay; C´´) corresponding brightfield view. Bar = 50 micrometer. (D) Expression analyses of co-cultures by RT-PCR. The endogenous murine *Cryaa* gene could be detected in P19 co-cultures. The endogenous lens-specific *CryF* transcript could be detected in NTERA-2/iPS and P19/iPS co-cultures, but also in P19 cells. Co^N^, co-culture of NTERA-2 and iPS; Co^P19^, co-culture of P19 and iPS; N, NTERA-2; P19, P19 cells; eye, positive control; -RT, without reverse transcriptase; H_2_0, no template. (E) Immunodetection of tdTomato protein. Co^N^, co-culture of NTERA-2 and iPS; Co^P19^, co-culture of P19 and iPS; N, NTERA-2; P19, P19 cells. (F) Expression analysis of co-cultures for key regulatory genes, Pax6 and Prox1, of lens differentiation by RT-PCR. CoN, co-culture of NTERA-2 and iPS; CoP19, co-culture of P19 and iPS; F, fibroblasts.

One day after seeding of the iPS cells on the different feeders, the stem cell medium was switched to a DMEM-based medium without LIF. The proliferation of iPS cells slowed down under these conditions. The cultures were split three days later and again seeded on the respective feeders. Around day 28 after seeding on feeders, the first tdTomato-positive cells were identified in the cultures with NTERA-2 and P19 feeders, but not in co-culture with STO cells. In some cases, individual cells expressed the reporter (Fig 6), in other cases the positive cells grouped to form lentoid bodies. The lentoid bodies also showed a changed light refraction in the brightfield view (Fig 6). At day 45 of the differentiation the cultures were used for molecular analyses, a mean of 5–10 lentoid bodies/well were counted at this time point. Expression analyses indicated that the co-cultures with NTERA-2 cells up-regulated the *tdTomato* and the endogenous *Cryaa* transcripts, but also other crystallin genes, like *Cryf*. Importantly, key regulator genes of lens differentiation, like *Pax6* and *Prox1* were also detected by RT-PCR (Fig 6).

## Discussion

Here, we generated a transgenic mouse line carrying an *alphaA crystallin*-promoter driven *tdTomato* reporter (cryTom), and generated an iPS cell line using a transposon-mediated approach. The generated iPS cell line was exploited in a proof-of-concept study for directed differentiation to lens cell lineage in a co-culture system. The cryTom mouse line was generated by *in ovo*-transposition, co-injecting the cryTom-transposon and a *piggyBac* helper plasmid. A detailed characterization of the cryTom mouse line suggested that the reporter faithfully mirrored the spatial and temporal expression pattern of the *Cryaa* gene. Induced PS cells could be derived from cryTom fibroblasts; upon exposing the cryTom iPS cells to a differentiation protocol, expression of the tdTomato reporter was resumed, thus allowing simple identification and vital recording of lentoid body growth *in vitro*.

For differentiation we used a novel co-culture system with the human NTERA-2, a committed neuronal precursor line [33] and P19, a murine embryonic carcinoma line [34]. Previously, *in vitro* differentiation to lens cells was achieved by co-culture [21], but also by supplementation of the culture media with defined growth factors, such as Noggin, FGF2 and Wnt-3a [2]. We hypothesized that the NTERA-2 and the P19 cell form “niches” for ectodermal differentiation of the cryTom iPS cells. It has been shown that the supplementation with retinoic acid can promote the expression of ectodermal characteristics of P19, but also of NTERA-2 [35,36]. This may be an approach to increase the directing effects of the feeder cells. Apparently, the spontaneous capabilities of mitotically inactivated NTERA-2 and P19 are sufficient to direct the differentiation of murine iPS cells into the lens cell lineage. The cryTom reporter construct allowed the unambiguous identification of onset of tdTomato expression as a faithful indicator of lens cell differentiation. Importantly, the detailed characterization of the spatio-temporal pattern of the cryTom reporter confirmed the exclusive expression in lens cell progenitors and mature lens cells.

The design of cell type specific-promoter sequences driving fluorescent reporters, such as EGFP or mCherry gained reasonable interest in genetics over the last years. Examples are the *Oct4* promoter-*EGFP* cassette [37], as well as a multitude of other constructs [38–40]. Recently, the development of hyperactive transposon systems made transposase-catalyzed gene integration an attractive alternative [41,42] to commonly employed random integration, or homologous recombination approaches. Here, we employed a piggyBac transposase catalyzed integration of the cryTom reporter *in ovo*, using a simplified plasmid microinjection technique in murine zygotes [29,43,44]. The main advantage of the transposase-catalyzed approach is that integration will take place at transcriptional permissive loci in the genome allowing for promoter-dependent expression [43,45]. Thus avoiding the generation of multiple founders and screening of those with appropriate expression patterns [46]. Indeed, from the first embryo transfer we obtained a single transgenic founder, which faithfully showed the expected phenotype. Importantly, the transposition of the cryTom reporter in zygotes supersedes the necessity to include any selection marker, such as an antibiotic resistance. It has been shown before that antibiotic selection markers and regulatory elements of secondary expression cassettes can exert massive effects on the primary expression construct, e.g. via promoter interference [47,48].

Importantly, the cryTom mice show a reduced light transmission through their lenses, a feature, which is also found in cataracts. Whether this is due to characteristics of the tdTomato protein itself, the accumulation of degraded products of tdTomato, or an unspecific interference of the ectopic protein with the highly ordered organisation of crystallins in the lens warrants further investigations. Here, we conducted a proof-of-principle study in murine cell cultures. The mouse model allowed to thoroughly investigate, whether the reporter expression faithfully reflect the temporal and spatial pattern of the endogenous gene. For human iPS cells the transduction *in ovo* is of course not a realistic option, however, the here gained evidence may allow to transpose existing human iPS cell lines with the reporter construct and still exploit the reporter for optimized differentiation approaches. The main advantage of the here described system is that it allows to optimizing the differentiation conditions. These optimized protocols may then be applied to human cells.

The present data show that it is possible to employ two different transposon systems, here PB and SB, to perform complex genetic modification. The current data support the notion that the cell type-specific reporter approach is instrumental for the development, validation and optimization of differentiation protocol of murine iPS cells into the lens cell lineage. The specific fluorescence of the tdTomato reporter will allow the development of semi-, or high throughput approaches for the rapid testing of media supplements. We speculate that the obtained knowledge can be translated to optimize lens cell differentiation of human iPS cells and thus to advance the growth of patient-specific lentoid bodies. Likely, the cell type-specific reporter approach is also adaptable for *in vitro* tracking of other cell types.

## Materials and Methods

### Ethics statement

Animals were maintained and handled according to the German laws for animal welfare, and genetically modified organisms. The experiments were approved by the authoritative external ethics committee of the LAVES (Niedersächsisches Landesamt für Verbraucherschutz und Lebensmittelsicherheit, AZ 33.9-42502-04-09/1718).

### Plasmid construction

A *Cryaa promoter-tdTomato* plasmid was gifted by T. Xu (Yale) [6]. The *Cryaa promoter-tdTomato* cassette was released by restriction with MluI and AflII and ligated in compatible sites between 5´and 3´ *piggyBac* ITR´s, resulting in pTTCryTom (cryTom) plasmid consisting of the *alphaA-crystallin* promoter, *tdTomato* cDNA and a *SV40* poly adenylation sequence, flanked by PB ITRs (S1 Fig). The PB transposase plasmid was described before [42], and essentially contained a cytomegalovirus, immediated early promoter driven hyperactive PB transposase cDNA. The SB reprogramming transposon carrying the murine cDNAs of *Oct4*, *Sox2*, *Klf4* and *cMyc* separated by sequences coding for self-cleaving 2A peptides, and the SB transposase helper plasmid were described before [32,49].

### Generation of PB transgenic founder mouse

The NMRI mice were bred and maintained in an air-conditioned animal quartier at 20°C and 60% humidity with 12 hour light and 12 h dark cycles. For zygote flushing, NMRI females of 5–6 weeks of age were superovulated by i.p. injection of 10 units PMSG and 10 units hCG in a 46–48 h interval. The treated animals were then mated with fertile males. Females with a copulation plug were identified the next day, sacrificed by CO_2_, and subsequently the isolated oviduct was flushed with M2-medium. Zygotes with two polar bodies were treated by cytoplasmic injection (CPI) of an equimolar mixture of pTTcryTom and PB helper plasmid [44,45]. A total of 20 treated zygotes were surgically transferred into the oviduct of a surrogate mother, resulting in the birth of 8 offspring of which one was transgenic for the cryTom transposon. The transgenic founder was used to establish a stable line by mating with a wild type animal. The offspring were phenotyped by whole body excitation with a green LED flood light, and images were recorded with a digital camera and an appropriate emission filter.

### Fluorescence microscopy

For fluorescence microscopy of cell cultures, a Zeiss Axiovert 35M microscope equipped with fluorescence optics was used. For specific excitation of tdTomato a filter block with excitation of 530–570 nm and emission of 590–610 nm were used. Alternatively, images were obtained by an Olympus BX 60 (Olympus, Hamburg, Germany) fluorescence microscope equipped with a high resolution digital camera (Olympus DP71).

For imaging of tissue biopsies an Olympus SZ16 stereomicroscope with epifluorescence optics was used. The light transmittance of lenses was also measures with the stereomicrosope. Therefore wildtype and transgenic lenses were isolated, and placed side by side under the stereozoom microscope. Normalized grey scale images were kept while illuminated from below. With the Olympus CellF software histograms (relative light transmission) of identically treated lenses (dotted lines in Fig 4A) were determined and plotted.

### Genotyping by PCR and Southern blotting

Southern blots and PCR reactions of genomic DNA were done according to standard procedures. In brief, for Southern blot detection of the transposon copies, the genomic DNA was digested with NcoI. Hybridisation with a *tdTomato* probe (1.6 kb fragment generated by BamHI and MfeI digest of *pTTcryTom*) resulted in constant internal fragments of ~ 0.5 and 0.7 kb and variable external fragment(s) of > 2.1 kb per integration. To assess for *PB* plasmid sequences, the blots were hybridized with a *PB* probe, generated by labelling the whole helper plasmid.

### Preparation of primary cell cultures

Fetuses of specific developmental stages were recovered from non-transgenic females mated with cryTom hemizygous males. The day of detection of a copulation plug was counted as day 0.5. Primary cells were derived from fetal tissue as described [49] and cultured in DMEM supplemented with 10% fetal calf serum and antibiotics. Fetal fibroblasts were cultured in high-glucose DMEM supplemented with 10% heat-inactivated fetal calf serum (PAA, Pasching, Austria), 2 mM L-glutamine, 1 mM sodium pyruvate, 1% non-essential amino acids, 0.05 mM β-mercaptoethanol, 100 U/ml penicillin, and 100 μg/ml streptomycin. Cells at passage 3 were used for electroporation with transposon plasmids. A Biorad electroporator with square wave function was used for electroporation. For feeder cells, primary murine embryonic fibroblasts (MEFs) were grown to subconfluency and inactivated with 10 μg/ml mitomycin C (Sigma) followed by thorough washings.

### iPS cell generation and cultivation

Induced pluripotent stem cells were cultured in ES cell medium consisting of DMEM/F12 supplemented with 20% knock-out serum replacement (Millipore), 1 mM L-glutamine, 0.1 mM non-essential amino acids (Gibco), 0.1 mM β-mercaptoethanol (Sigma), 100 U/ml penicillin, 100 μg/ml streptomycin, and 1000 units/ml LIF (Santa Cruz) in a humidified atmosphere consisting of 5% CO_2_ in air at 37°C.

The iPS cells were maintained on gelatinized plates, or plates seeded with inactivated MEFs feeders and enzymatically (trypsin/EDTA) subpassaged every second or third day. For gelatinization, the intended culture dishes were wetted with sterile 1% gelatin in PBS and allowed to dry immediately before sub-passaging. Alternatively, the iPS cells were passaged on MEF feeders seeded the day before.

### *In vitro* differentiation assays

NTERA-2 [33] and P19 [34] cells were obtained from the Deutsche Sammlung für Mikroorganismen (DSMG, Braunschweig) and cultured in high-glucose DMEM medium supplemented according to the description in “Preparation of primary cell culture”. The cultures were split in 1:6 to 1:8 ratios in 2–3 day intervals. STO cells were treated identically. For mitotic inactivation, NTERA-2, P19 and STO cells were grown to subconfluency, respectively, and incubated in fresh medium containing 10 μg/ml mitomycin C for 3 hours followed by thorough washings with PBS.

For ectodermal differentiation, iPS cells were trypsinized and re-suspended in regular ES cell medium for generation of embryoid bodies (EBs). To induce EB formation, the hanging-drop method was used and drops of 20 μl containing 600 cells were pipetted onto the lids of 10 cm cell culture dishes and incubated at 37°C for three days. EBs were washed off the plate with PBS and transferred to 6-well plates seeded with inactivated NTERA-2, P19 or STO cells, respectively. The stem cell medium containing LIF was replaced one day after EB seeding against a DMEM medium containing 1% FCS, 2 mM L-glutamine, 1 mM sodium pyruvate, 1% NEAA, 0.05 mM ß-mercaptoethanol, 100 U/ml penicillin and 100 μg/ml streptomycin (without LIF). The cultures were inspected in regular intervals of 3–5 days for the appearance of tdTomato-positive cells. As controls, NTERA-2, P19, STO and iPS cells were cultured individually.

### Alkaline phosphatase staining

Cells were fixed with 4% formaldehyde, washed with Tris-buffered saline (with 0.1% Tween-20) and stained with AP staining solution [32].

### Reverse transcription-PCR

Total RNA was prepared using TriReagent (Ambion, Germany) according to the manufacturer´s instructions. Isolated total RNA from cell samples was treated with RNase-free DNase (1 U/μg RNA) (Epicentre Biotechnologies, Madison, WI) and 0.5 μg was used for cDNA synthesis. Reverse transcription (RT) was performed in a 20 μl volume consisting of 4 μl of 10x RT buffer (Invitrogen), 4 μl of 50 mM MgCl_2_ (Invitrogen), 4 μl of 10 mM dNTP solution (Bioline), 2μl (20 Units) of RNAsin (Applied Biosystems), 2 μl (50 Units) of MMLV reverse transcriptase (Applied Biosystems) and 2 μl hexamers (50 μM) (Applied Biosystems). The samples were incubated at 25°C for 10 minutes for primer annealing and then incubated at 42°C for 1 hour. Finally, the samples were heated to 95°C for 5 minutes. The cDNA was diluted 1:5 and 2 μl (10 ng) were used for PCR amplification. PCR program: activation of the Taq Polymerase for 10 min at 95°C followed by 40 cycles of 95°C for 15 s and 60°C for 1 min. Primer sequences are listed in S1 Table. As control, the housekeeping genes *Gapdh* or *Papola* were amplified.

### Western blotting

Finely grinded tissues and cells were extracted in RIPA buffer, and 10 microgram of protein per slot was separated on 12% SDS-PAGE gel, blotted to PVDF membrane, blocked in 5% non-fat milk powder and probed with a rabbit polyclonal antibody against mCherry, which is cross-reactive with other red fluorophore variants, such as tdTomato (Thermo) in 1:1000 dilution. This was followed by a secondary anti-rabbit antibody in 1:10 000 dilution (Sigma-Aldrich). For detection of the endogenous alphaA crystallin an antibody from Santa Cruz (alphaA crystallin, sc-28306) was use at 1: 2000 dilution. For detection an ECL+ kit (GE Healthcare) and an image acquisition system (Vilber Lourmat, Fusion SL 3500) were used.

### Bioinformatic searches

Genevestigator server (www.genevestigator.com) was used to examine expression data of *Cryaa*. Genevestigator summarises DNA array data of several independent studies indicating highest *Cryaa* mRNA level in the eye lens, low levels in eyecup, ciliary body and retina, and undetectable levels in other organs.

## Supporting Information

S1 Fig Generation of cryTom founder. (A) Scheme of the cryTom transposon. An expression cassette of the alphaA crystallin (Cryaa) promoter driving tdTomato-cDNA and a poly adenylation sequence is flanked by 5´ and 3´-ITR´s of PB. For generation of transposon mice, the cryTom transposon was co-injected together with a PB expression plasmid (helper plasmid) into the cytoplasm of murine zygotes. N, NcoI site; dotted line, labeled probe for Southern blotting. Drawing not at scale. (B) Founder animal shown under daylight conditions, B´) specific excitation of tdTomato and B´´) overlay of both images. The animal was imaged, while sleeping under a stereomicroscope equipped with epifluorescence. (C) Newborn F1-offspring (two transposon pups and a non-transgenic littermate) shown under specific excitation of tdTomato. Scale bars = 1 cm. (D) Southern blotting of founder, F1 and F2 offspring. The design of the Southern blot predicts two internal fragments of constant size, and a flanking fragment depending of the next neighbouring NcoI site in the genome.
(DOCX)

S1 Table Primers used in RT-PCR. (DOCX)
