# Supplementary material for: Differentiation of Induced Pluripotent Stem Cells to Lentoid Bodies Expressing a Lens Cell-Specific Fluorescent Reporter
Source: PLoS One. 2016 Jun 20;11(6):e0157570. doi: 10.1371/journal.pone.0157570 (PMC4913943; doi:10.1371/journal.pone.0157570)
Supplement: S1 Fig — (A) Scheme of the cryTom transposon. An expression cassette of the alphaA crystallin (Cryaa) promoter driving tdTomato-cDNA and a poly adenylation sequence is flanked by 5´ and 3´-ITR´s of PB. For generation of transposon mice, the cryTom transposon was co-injected together with a PB expression plasmid (helper plasmid) into the cytoplasm of murine zygotes. N, NcoI site; dotted line, labeled probe for Southern blotting. Drawing not at scale. (B) Founder animal shown under daylight conditions, B´) specific excitation of tdTomato and B´´) overlay of both images. The animal was imaged, while sleeping under a stereomicroscope equipped with epifluorescence. (C) Newborn F1-offspring (two transposon pups and a non-transgenic littermate) shown under specific excitation of tdTomato. Scale bars = 1 cm. (D) Southern blotting of founder, F1 and F2 offspring. The design of the Southern blot predicts two internal fragments of constant size, and a flanking fragment depending of the next neighbouring NcoI site in the genome. (DOCX) [file pone.0157570.s001.docx]

**Supplementary information**


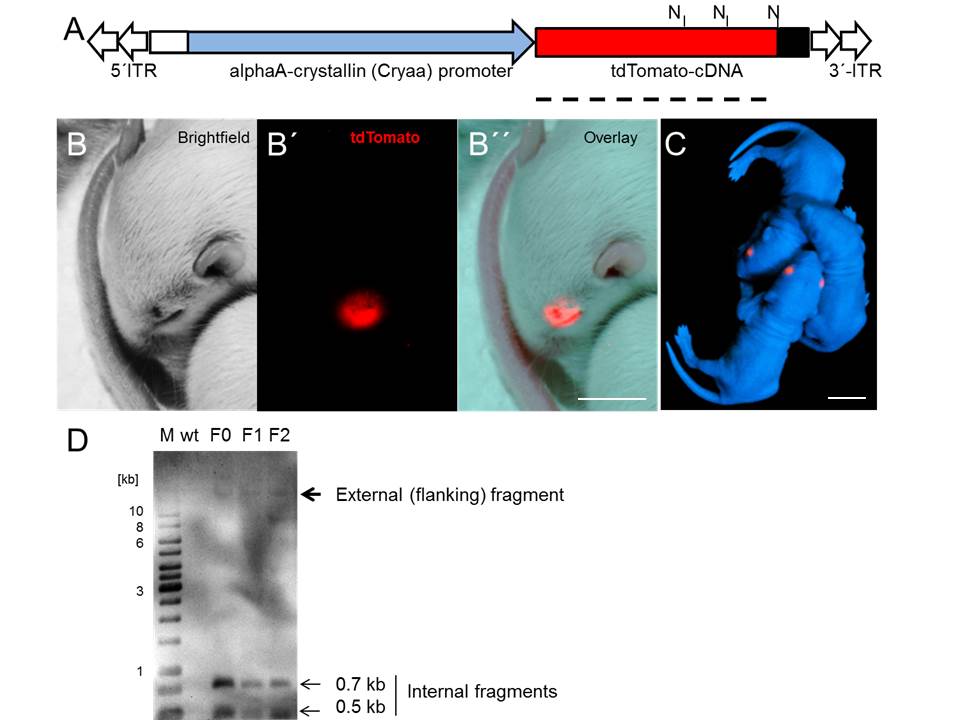


**S1 Fig. Generation of cryTom founder**

A) Scheme of the cryTom transposon. An expression cassette of the alphaA crystallin (Cryaa) promoter driving tdTomato-cDNA and a poly adenylation sequence is flanked by 5´ and 3´-ITR´s of PB. For generation of transposon mice, the cryTom transposon was co-injected together with a PB expression plasmid (helper plasmid) into the cytoplasm of murine zygotes. N, NcoI site; dotted line, labeled probe for Southern blotting. Drawing not at scale.

B) Founder animal shown under daylight conditions, B´) specific excitation of tdTomato and B´´) overlay of both images. The animal was imaged, while sleeping under a stereomicroscope equipped with epifluorescence.

C) Newborn F1-offspring (two transposon pups and a non-transgenic littermate) shown under specific excitation of tdTomato. Scale bars = 1 cm.

D) Southern blotting of founder, F1 and F2 offspring. The design of the Southern blot predicts two internal fragments of constant size, and a flanking fragment depending of the next neighbouring NcoI site in the genome.
