# Supplementary material for: Differentiation of Induced Pluripotent Stem Cells to Lentoid Bodies Expressing a Lens Cell-Specific Fluorescent Reporter
Source: PLoS One. 2016 Jun 20;11(6):e0157570. doi: 10.1371/journal.pone.0157570 (PMC4913943; doi:10.1371/journal.pone.0157570)
Supplement: S1 Table — (DOCX) [file pone.0157570.s002.docx]

**Supplementary information**

**S1 Table. Primers used in RT-PCR**

| Gene | Primers | Annealing temp (˚C) | Product size (bp) |
| --- | --- | --- | --- |
| Endo_mOct4 | 5´-ATG AAA GCC CTG CAG AAG GAG CTA GAA C | 55 | 352 |
|  | 5´-TCT CTA GCC CAA GCT GAT TGG CGA TGT G |  |  |
| Endo_mSox2 | 5´-TAG AGC TAG ACT CCG GGC GAT GA | 55 | 274 |
|  | 5´-TTG CCT TAA ACA AGA CCA CGA AA |  |  |
| Endo_mKlf4 | 5´-GCG AAC TCA CAC AGG CGA GAA ACC | 55 | 695 |
|  | 5´-TCG CTT CCT CTT CCT CCG ACA CA |  |  |
| Endo_m c-Myc | 5´-TGA CCT AAC TCG AGG AGG AGC TGG AAT C | 55 | 171 |
|  | 5´-AAG TTT GAG GCA GTT AAA ATT ATG GCT GAA GC |  |  |
| Endo _mUtf1 | 5´-GGA TGT CCC GGT GAC TAC GTC TG | 57 | 345 |
|  | 5´-GGC GGA TCT GGT TAT CGA AGG GT |  |  |
| Endo _mRex1 | 5´-ACG AGT GGC AGT TTC TTC TTG GGA | 57 | 286 |
|  | 5´-TAT GAC TCA CTT CCA GGG GGC ACT |  |  |
| Endo_ mNanog | 5´-AGG GTC TGC TAC TGA GAT GCT CTG | 55 | 355 |
|  | 5´-CAA CCA CTG GTT TTT CTG CCA CCG |  |  |
| Gapdh | 5´-CAAGGTCATCCATGACAACTTTG | 55 | 496 |
|  | 5´-CTACAGCAACAGGGTGGTGGAC |  |  |
